# Initiation of intravitreal aflibercept injection treatment in patients with diabetic macular edema: a review of VIVID-DME and VISTA-DME data

**DOI:** 10.1186/s40942-016-0041-z

**Published:** 2016-07-11

**Authors:** Focke Ziemssen, Patricio G. Schlottman, Jennifer I. Lim, Hansjürgen Agostini, Gabriele E. Lang, Francesco Bandello

**Affiliations:** 1 Centre for Ophthalmology, Eberhard-Karls University of Tuebingen, Schleichstrasse 12, 72076 Tübingen, Germany; 2 Organizacion Medica de Investigacion, Uruguay 725 PB, C1015ABO Buenos Aires, Argentina; 3 University of Illinois at Chicago, Illinois Eye and Ear Infirmary, 1855 W. Taylor Street, M/C 648, Chicago, IL 60612 USA; 4 Eye Center, Medical Center, Faculty of Medicine, University of Freiburg, Killianstr. 5, 79106 Freiburg, Germany; 5 Division of Medical Retina and Laser Surgery, Department of Ophthalmology, University Eye Hospital Ulm, Prittwitzstr. 43, 89075 Ulm, Germany; 6 Department of Ophthalmology, IRCCS San Raffaele Scientific Institute, University Vita-Salute San Raffaele, Via Olgettina 60, 20132 Milan, Italy

**Keywords:** Aflibercept, Anti-VEGF, Diabetic macular edema, Upload, Treatment initiation, Loading dose

## Abstract

**Background:**

Diabetic macular edema (DME) shows a gradual and sustained functional and morphologic response to anti-vascular endothelial growth factor (VEGF) drugs, but the optimal schedule for initiation of anti-VEGF therapy is not known. This study evaluates the treatment response behavior of DME in the Phase 3 trials of intravitreal aflibercept, with 5 initial intravitreal aflibercept injections (IAI), 2 mg every 4 weeks (2q4), in the upload phase.

**Methods:**

This post hoc pooled analysis of the VISTA-DME (NCT01363440) and VIVID-DME (NCT01331681) trials evaluated the change in best-corrected visual acuity (BCVA) and central retinal thickness (CRT) during the upload phase, using pooled data from both IAI treatment groups [2q4 and 2 mg every 8 weeks (2q8)]. The mean visit-to-visit change in BCVA and CRT, and the respective rate of gainers and losers was calculated for each successive visit. A secondary analysis compared the visit-to-visit change in BCVA between the 2q4 and 2q8 treatment arms during the upload period and the first year treatment period.

**Results:**

The majority of eyes showed a continuing improvement of BCVA after the first IAI. The proportions of eyes gaining BCVA (≥5 letters) at each visit compared with the previous visit during the IAI 2q4 upload phase were 60 (4-weeks), 19 (8-weeks), 16 (12-weeks), 15 (16-weeks), and 14 % (20-weeks). In contrast, the proportions of eyes losing BCVA (≥5 letters) were 3 (4-weeks), 7 (8-weeks), 7 (12-weeks), 9 (16-weeks), and 8 % (20-weeks), respectively. The odds of BCVA (≥5 letters) gain/loss exceeded 1.7 at each visit (range 1.7–20). Overall, the proportion of patients with BCVA gain ≥5 letters at week 20 (compared with baseline) was 76 and 80 % in the 2q4 and 2q8 groups, respectively. The proportions of eyes showing a visit-to-visit decrease in CRT of ≥30 µm during the first 5 IAI were 77 (4-weeks), 27 (8-weeks), 21 (12-weeks), 17 (16-weeks), and 12 % (20-weeks). In the secondary analysis, the BCVA outcomes were similar for the 2q8 and 2q4 treatment arms.

**Conclusions:**

The data presented here are consistent with continual functional and anatomic improvement following the fourth and fifth initial 2q4 injections, suggesting that an intensive and sufficiently long upload may be beneficial.

*Trial registration* VIVID-DME: Clinicaltrials.gov: NCT01331681; VISTA-DME: Clinicaltrials.gov: NCT01363440

## Background

Intravitreal anti-vascular endothelial growth factor (VEGF) medications are recognized for improving visual outcomes and decreasing macular fluid in patients with diabetic macular edema (DME) [1–3]. In contrast to other retinal diseases, the response has been shown to be more “gradual”—the curves of best-corrected visual acuity (BCVA) and central retinal thickness (CRT) more slowly approach their values of maximal improvement during the treatment every 4 weeks (Table 1; [4–12]), resulting in a long first phase of improvement [1]. Importantly, long-term studies have shown that the favorable outcome can be maintained for at least 3–5 years, with a significantly reduced number of treatments during the later follow-up [6].

**Table 1:** Treatment initiation and continuation regimens of Phase 3 randomized controlled trial of anti-VEGF in DME

Randomized controlled trial	Initiation and continuation regimen	Time to maximum mean BCVA*
RIDE/RISE NCT00473382 NCT00473330	Fixed q4 intervals ranibizumab (no reduction before week 156 in the open-label extension) [4]	Week 88/week 92
RESTORE NCT00687804	Initial 3 q4 intervals followed by PRN (protocol-driven) ranibizumab retreatment [5, 6]	Week 40
RELIGHT NCT01257815	Initial 3 q4 intervals followed by PRN (protocol-driven) ranibizumab retreatment [7]	Week 24
REVEAL NCT00989989	Initial 3 q4 intervals followed by PRN (protocol-driven) ranibizumab retreatment [8]	Week 32
DRCR protocol I NCT00445003	Initial 4 q4 intervals ranibizumab followed by protocol-driven ranibizumab retreatment [9]	Week 232
DRCR.net protocol T NCT01627249	Aggressive retreatment protocol for IAI, bevacizumab, and ranibizumab prior to 24-week visit with q4 intervals [10]	Week 32, week 52
VIVID/VISTA NCT01363440 NCT01331681	Initial 5 q4 intervals IAI followed by 2q8 or 2q4 intervals [11]	Week 44
BOLT EUDRACT2007-000847-89	Initial 3 q6 intervals bevacizumab (initially combined with laser) followed by PRN (protocol-driven) bevacizumab retreatment [12]	Week 100

q4, every 4 weeks

* Within controlled/core study phase

What is less clear at this point, however, is the optimal dosing schedule for initiation of anti-VEGF therapy in DME. Given that the disease is characterized by high levels of intravitreal VEGF [9], there is clearly a rationale for aggressive initial therapy; however, specific initiation algorithms have not been evaluated as the independent variable within Phase 3 clinical trials, and indirect comparisons-across study populations or drugs used-are of limited value. The 36-month results from the DA VINCI study suggest that 5 initial 2 mg intravitreal aflibercept injections (IAI) may be beneficial, whereas the 2-year Protocol T results show that individualized initiation schemes that led to more than 5 doses for initiation resulted in a favorable outcome [13, 14]. There are currently no clearly defined predictors for identifying patients with DME who are in need of either an intensified or less intensive initial treatment with anti-VEGF therapy.

A worldwide comparison showed a widespread diversity of initiation recommendations for anti-VEGF treatment in DME, ranging from series of 3, 4, or 5 to patterns of 4 + 2 injections [2, 15]. Some national guideline committees refrain from giving any specific guidance on the best initiation strategy [16]. Other recommendations define the dosing scheme in relation to the drug used (according to the study protocols) [16–19]. Still other societies suggest a minimum of 4 initial injections in accordance with the DRCR.net treatment protocol [20, 21], which was also adopted in a slightly modified form in a recent head-to-head comparison of anti-VEGF drugs in the treatment of DME [9]. In the DRCR study, the majority of patients received an intensive upload (6 monthly treatments initially) that was not mandated, suggesting the beneficial effects of a more intense initial schedule [2], with decreased dosing requirement seen in Year 2 [15].

Aflibercept is a 115 kDa fusion protein composed of extracellular domains from human VEGF receptors 1 and 2, and has been shown to inhibit both VEGF-A and placental growth factor (PGF) [22]. Preclinical studies have shown a long duration of action and high binding affinity to VEGF-A.

Given the variability in guidance and incomplete evidence base, it may be valuable to examine more closely the efficacy data for a well-studied initiation protocol, IAI 2 mg every 4 weeks (2q4) for 5 doses, and in particular to ascertain if there is evidence for continued improvement following the fourth and fifth injections in the series. This post hoc analysis evaluates the visit-to-visit visual acuity and CRT responses to the initial 5 IAI given in the Phase 3 trials. This detailed look at the treatment response during the upload phase provides greater clarity regarding the responsiveness of DME to this upload strategy.

## Methods

VISTA and VIVID were 2 similarly designed, double-masked, randomized, active-controlled, 148-week, Phase 3 trials. VISTA was conducted across 54 sites in the United States, and VIVID took place in 73 sites across Europe, Japan, and Australia. Both VISTA and VIVID were conducted in compliance with the International Conference on Harmonisation guidelines and the Health Insurance Portability and Accountability Act of 1996. The study protocol has been described in detail [11]. Briefly, patients with central-involved DME (defined as retinal thickening involving the 1-mm central area as measured by optical coherence tomography) were eligible for enrollment if BCVA was between 73 and 24 letters (20/40–20/320 Snellen equivalent) in the study eye. After 5 initial doses at 4-week intervals (IAI 2q4), IAI eyes received treatment in accordance with the protocol [2q4, 2 mg every 8 weeks (2q8)].

This study was a post hoc analysis of the initial response to IAI treatment in the VISTA (NCT01363440) and VIVID (NCT01331681) trials, with BCVA performed per a defined protocol at every visit, OCT performed with a SD-OCT at every visit, and read by independent reading centers. Pooled data included all patients in the full analysis set from both studies, defined as all randomized patients who received any investigational product and had a baseline and at least 1 post-baseline assessment of BCVA. We evaluated the change in the BCVA and the reduction in CRT from the immediately preceding visit to the immediately following visit for each of the first 5 IAI 2q4 doses. This analysis was done for the pooled data from the 2q4 and 2q8 groups of VIVID and VISTA, as both groups received the same number of treatments during the upload phase. Therefore, the analysis reflects the approved dosing in both the European Medicines Agency and US Food and Drug Administration labels.

When assessing the visit-to-visit change, a threshold of 5 Early Treatment Diabetic Retinopathy Study (ETDRS) letters and 30 µm was chosen as the cutoff for defining categorical change (for the responder analysis). These cutoff values were chosen, as lower thresholds would be more vulnerable to measurement variability. The change in retinal thickness of 30 µm corresponds to approximately one-tenth of the total thickness. The proportion of gainers and losers and the ratio of gainers/losers were calculated for each of the first 5 dosing intervals.

In addition, as a secondary analysis, the proportion of patients who gained ≥5 ETDRS letters from baseline was compared between the pooled 2q8 and the pooled 2q4 treatment arms of VIVID and VISTA by for each follow-up visit through week 72. Finally, we summarized the 100-week safety data for these 2 pooled treatment groups in order to provide a more complete comparison.

## Results

### Upload analysis

**Fig. 1:**
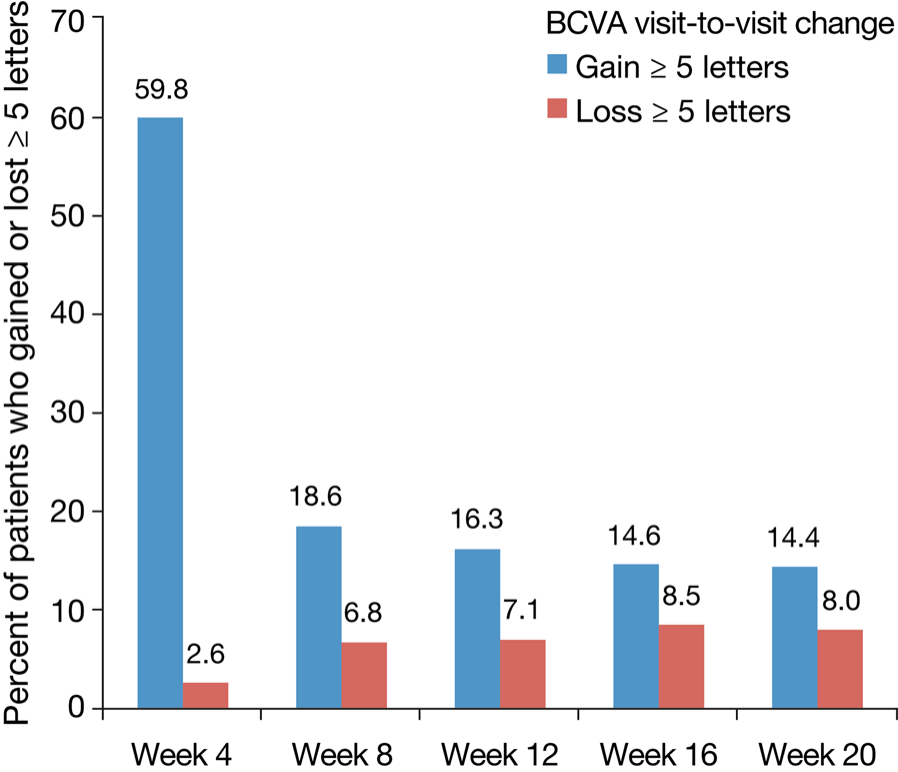
Percentage of patients who gained or lost 5 letters; change of BCVA is always compared with the previous visit (n = 576)

**Fig. 2:**
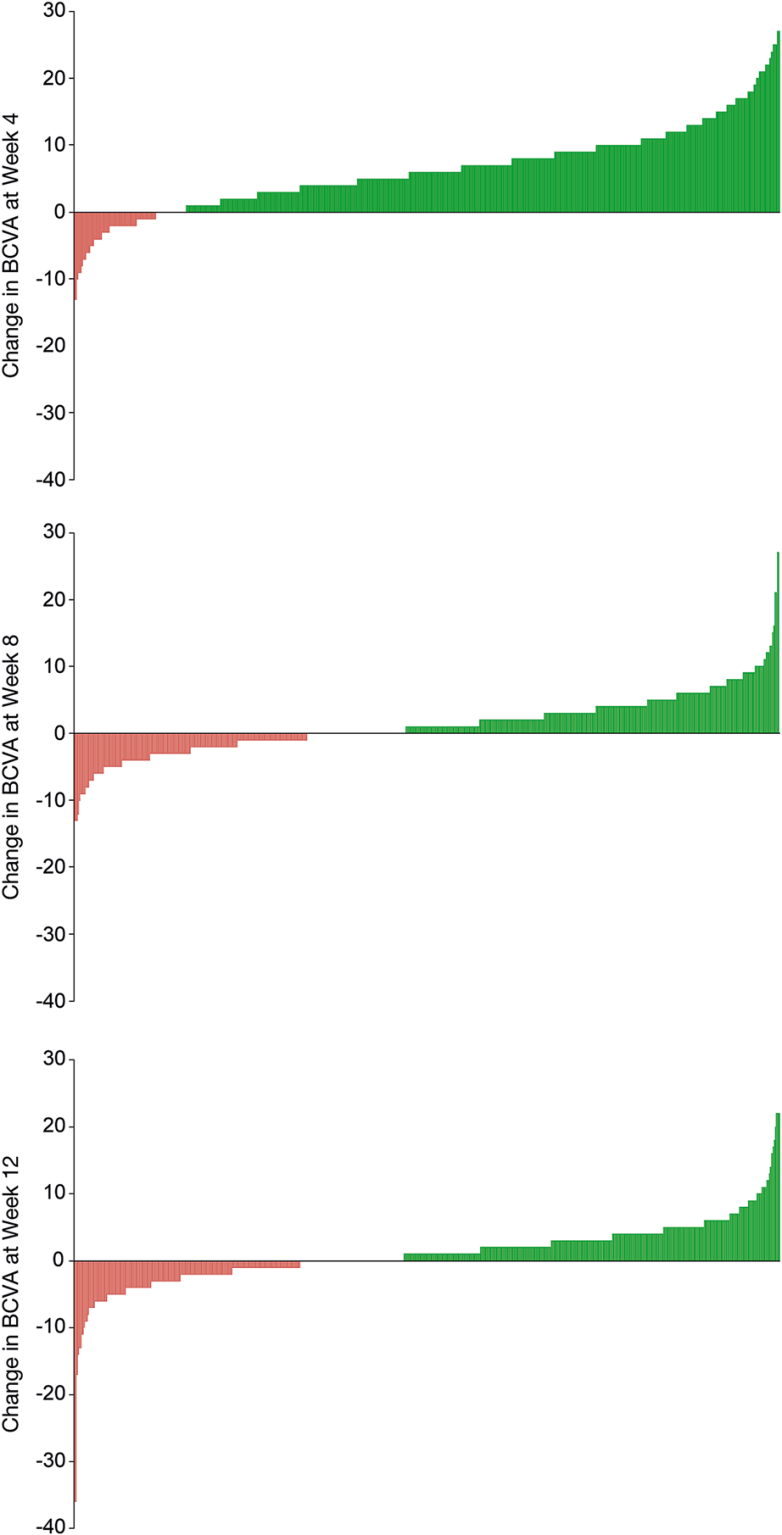
Waterfall plots of BCVA change at weeks 4, 8, and 12

There were 576 patients in the pooled full analysis set. The highest percentage of patients attaining visual gain of at least 5 letters was seen 4 weeks after the first IAI (59.8 %, Fig. 1). The lowest percentage of patients experiencing visual acuity loss of at least 5 letters was also seen 4 weeks after the first IAI (2.6 %, Fig. 1). At 4 weeks following the fifth IAI, 14.4 % of patients (1 in 7) had BCVA gain ≥5 letters, while 8.0 % had BCVA loss of ≥5 letters (Fig. 1). The ratio of gainers to losers after the first IAI was 23.0; it decreased to 2.7 after the second IAI and remained around 2 subsequently (2.3, 1.7, and 1.8 after the third, fourth and fifth IAI, respectively). Thus there were more gainers than losers following all 5 treatments of initial 2q4 dosing (Figs. 1, 2).

**Fig. 3:**
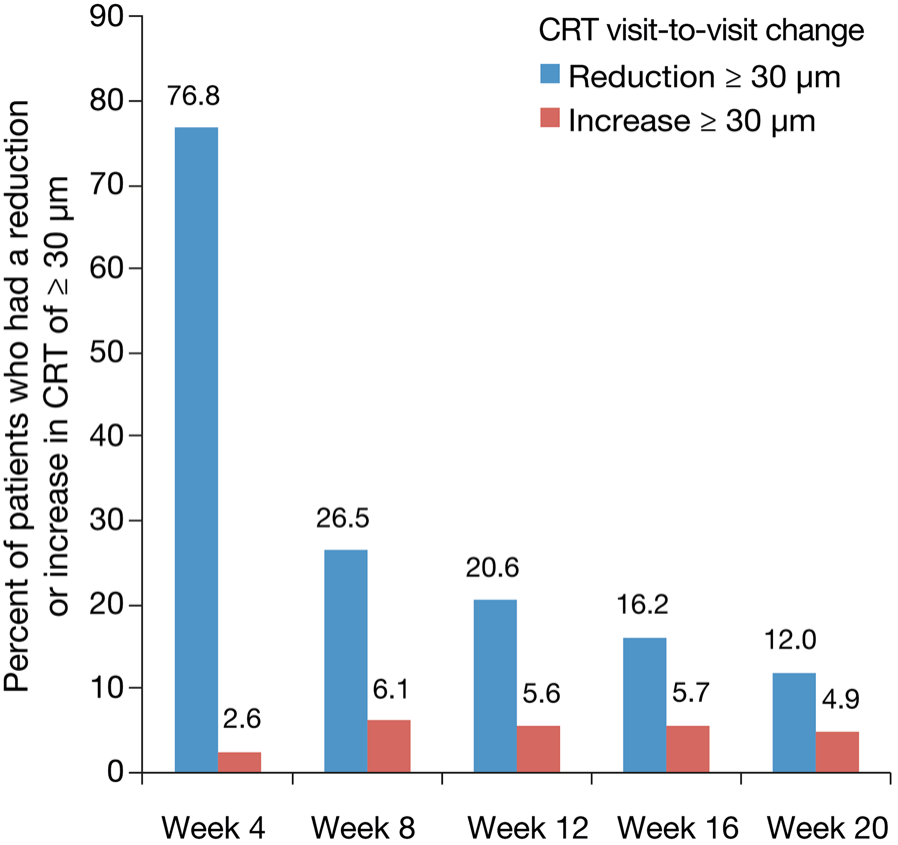
Visit-to-visit change in CRT: percentage of patients who had a reduction or increase in CRT of ≥30 µm (n = 576)

The anatomic response of these patients was supported by the CRT measurements showing similar proportions of reductions and increases (Fig. 3). The highest percentage of patients attaining reduction in CRT of ≥30 µm was seen 4 weeks after the first IAI (76.8 %, Fig. 3), as was the lowest percentage of patients with CRT increase (2.6 %). At 4 weeks following the fifth IAI, 12.0 % of patients (about 1 in 8) had CRT reduction of ≥30 µm whereas 4.9 % had CRT increase of ≥30 µm. Thus, the ratio of gainers to losers after the first IAI was 29.5; it decreased after each subsequent IAI but was still 2.4 following the fifth IAI (Fig. 3).

### Secondary analyses

**Fig. 4:**
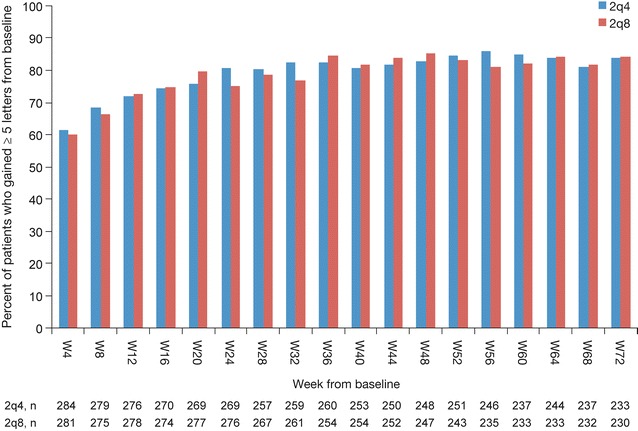
Percentage of patients who gained ≥5 letters from baseline, by visit

The mean change in BCVA from baseline to each visit up to 72 weeks for the pooled 2q8 and the pooled 2q4 arms of VIVID and VISTA is shown in Fig. 4. There is continuous improvement through week 20, representing the 4-week follow-up following the fifth IAI, with 75.8 % of patients in the 2q4 and 79.8 % of patients in the 2q8 groups gaining ≥5 letters at week 20. During further treatment for 1 year, during which the 2q4 group received treatment every 4 weeks and the 2q8 group received treatment every 8 weeks, no large shifts of these proportions were observed for either dosage arm and the values were similar at each time point.

### Safety

In both VISTA and VIVID, overall incidence of ocular and nonocular adverse events (AEs) was similar across treatment groups [11]. Over the course of the 100-week treatment period, there were no cases of endophthalmitis in any eyes treated with IAI in either study. The most common ocular AEs were conjunctival hemorrhage [99/291 (34.0 %) IAI 2q4, 81/287 (28.2 %) IAI 2q8, and 180/578 (31.1 %) all IAI], cataract [36 (12.4 %), 31 (10.8 %), 67 (11.6 %)], and eye pain [34 (11.7 %), 28 (9.8 %), 62 (10.7 %)]. The most common non-ocular AEs, respectively, were hypertension [67 (23.0 %), 63 (22.0 %), 130 (22.5 %)], nasopharyngitis [49 (16.8 %), 51 (17.8 %), 100 (17.3 %)], and urinary tract infection [23 (7.9 %), 36 (12.5 %), 59 (10.2 %)] [11]. Overall rates of Anti-Platelet Trialists’ Collaboration-defined arterial thromboembolic events, including vascular deaths, were low and comparable across the treatment groups. The incidence of death in the IAI 2q4, IAI 2q8, and control groups was 5.2, 2.6, and 1.9 %, respectively, in VISTA, and 2.9, 4.4, and 0.8 % in VIVID [11]. Overall, the AE profile over the 100 weeks of the study was consistent with the known safety profile of IAI.

## Discussion

This analysis illustrates the dynamic nature of the improvements in visual acuity during the upload phase, or first 20 weeks, of IAI 2q4 treatment. Specifically, in this analysis of pooled VIVID/VISTA data, we have shown that even though most shifts toward better visual acuity already occur after the very first injection, there are still considerably more BCVA gainers than losers following each of the subsequent 4 treatments during the IAI 2 q4 upload phase. This response in vision of patients with DME is paralleled by the CRT measurements showing similar proportions of reductions and increases. Therefore, these data support the potential benefit of at least 5 initial IAI 2q4 as an upload treatment regimen.

We also showed that over the course of the subsequent 52 weeks, during which one treatment arm received IAI 2q8 and the other continued to receive IAI 2q4, the visual acuity efficacy results, as well as the safety results, were highly similar. These data indicate that the longer-term outcome appears to be similar with less frequent dosing after the upload phase.

The data presented here are consistent with what has been observed in other important studies of anti-VEGF treatment for DME (Table 1). As these studies have shown, the rise in visual acuity in DME is gradual and peak visual acuity is only established after 6–9 months or longer [23]. This suggests that the initial treatment phase is particularly critical for final treatment success [10, 24, 25]. Although caution must be exercised when comparing across studies, there seems to be a pattern demonstrated in studies with a less intense/rigorous treatment initiation that outcomes may not be as good as outcomes in studies with a more rigorous and intense initiation scheme (e.g., RESTORE, Protocol I) [5, 6, 10].

With regard to IAI, the Phase 2 DA VINCI study showed for the first time that outcomes are similar with anIAI 2q8 dosing schedule compared with 2q4 or PRN dosing, and the requirement for monitoring can be reduced [13]. However, the study also suggests that visual acuity is less stable in the early treatment phase and that a more intensified treatment initiation could be beneficial. Hence, in the Phase 3 studies for IAI, an additional dose was added at week 12, resulting in a sequence of 5 initial IAI 2q4 before extending the treatment interval to 2q8 in one of the treatment arms. In summary, the available data suggest that visual acuity in DME patients may be impacted by the number of initial treatments during the upload phase. Our analysis of the pooled data from the upload phase of VIVID and VISTA suggests that this vulnerable phase may best be managed with the intensified dosing regimen of 5 initial consecutive doses at 4-weeks intervals.

The implementation of an initial treatment series can minimize the risk of under-treatment. In particular for patient education, the concept of a consistent initiation series can enhance the attendance at follow-up visits and adherence to the therapy [26]. If patients understand the need for a strict retreatment, the chance of reaching the maximum BCVA potential may be higher. A series of treatments can reduce organizational efforts and might minimize the impact of potential diagnostic errors (optical coherence tomography criteria) at decision points. Moreover, the administration of 5 consecutive IAIs does not preclude any additional interim fundus or safety examination, if considered necessary on a case-by-case basis.

This analysis has important strengths. First, it is based on a large data set from 2 pivotal randomized controlled trials of patients treated with the identical upload regimen. Second, it fills a gap in the field insofar as this particular regimen has not been well represented in previous analyses. Third, longer-term efficacy and safety strengthen the finding that following this particular upload regimen, outcomes remain similar despite less frequent IAI dosing (2q8 vs. 2q4).

This analysis also has several limitations. Only 1 loading dose phase is analyzed; thus, no direct conclusions can be drawn about the comparative efficacy of this initial treatment schedule with any other treatment scheme. In addition, the duration of action of each injection is not necessarily limited to the first 4 weeks following the injection, as the long-term experience suggests a vascular remodeling in DME under anti-VEGF therapy [9]. Thus, the visit-to-visit change cannot be solely attributedto the most recent injection, but also to the previous treatment, as well asother confounding factors. Although the simple responder analysis of the visit-to-visit change, as carried out in this study and described in this article, does not allow for predicting or assessing the response of an individual patient, it is supportive of an intensive treatment start, which is in line with observations that have been made in other studies.

## Conclusions

This post hoc analysis has demonstrated continuous improvement during the treatment initiation schedule of 5 IAI at 4-weeks intervals implemented in the VIVID and VISTA randomized clinical trials. These data support the potential benefit of this pragmatic treatment initiation strategy in the treatment of DME.

## Abbreviations

2q4: 2 mg given every 4 weeks (intravitreal aflibercept injections)
2q8: 2 mg every 8 weeks (intravitreal aflibercept injections)
BCVA: best-corrected visual acuity
CRT: central retinal thickness
DME: diabetic macular edema
ETDRS: Early Treatment Diabetic Retinopathy Study
IAI: intravitreal aflibercept injection
OCT: optical coherence tomography
SD-OCT: spectral-domain optical coherence tomography
VEGF: vascular endothelial growth factor
